# Drug concentrations in the serum and cerebrospinal fluid of patients treated with cefoperazone/sulbactam after craniotomy

**DOI:** 10.1186/s12871-015-0012-1

**Published:** 2015-03-13

**Authors:** Qiang Wang, Yuanxing Wu, Biyao Chen, Jianxin Zhou

**Affiliations:** 1 Intensive Care Unit, Beijing Tiantan Hospital, Capital Medical University, Beijing, 100050 PR China; 2 Department of Emergency, Beijing Tiantan Hospital, Capital Medical University, Beijing, 100050 PR China

**Keywords:** Cefoperazone, Sulbactam, Concentration, Serum, Cerebrospinal fluid

## Abstract

**Background:**

To identify changes in cefoperazone/sulbactam penetration into cerebrospinal fluid (CSF) after craniotomy and to investigate preliminarily whether cefoperazone/sulbactam CSF concentration can reach therapeutic level when administered intravenously after neurosurgical operation.

**Methods:**

Neurosurgical patients with an indwelling ventricular drainage pipe who received prophylactic cefoperazone/sulbactam for the treatment of intracranial infection were received a cefoperazone/sulbactam 2:1, 3.0-g infusion for 3 hours every 6 hours for 24 h. Venous blood and CSF specimens were collected to determine cefoperazone/sulbactam concentrations.

**Results:**

The cefoperazone and sulbactam concentrations in serum were highest at the second hour (237.54±336.72 mg/L and 66.52±80.38 mg/L, respectively) and then decreased. The cefoperazone and sulbactam concentrations in CSF were highest at the 4th hour (39.22±75.55 mg/L and 6.24±8.35 mg/L, respectively) and then decreased. CSF penetration measured by the ratio of peak concentrations (CSF/serum) was 8.6%±7.2% for cefoperazone and 13.5%±11.9% for sulbactam, CSF penetration measured by the ratio of trough concentrations (CSF/serum) was 13.4%±5.3% for cefoperazone and 106.5%±87.5% for sulbactam. CSF penetration represented by the ratio of area under the curve (AUC) of CSF and serum was 14.5% for cefoperazone and 22.6% for sulbactam. Neurosurgical impairment of the blood–brain barrier may improve the CSF penetration of these drugs, but it is difficult to reach the MIC_90_ of resistant bacteria. If single intravenous administration time was extended to 3 hours, the serum concentrations of drugs were able to meet the PK/PD standard (T> MIC%> 50%) for treating common, highly resistant bacteria.

**Conclusions:**

The CSF penetration of cefoperazone/sulbactam may be enhanced after neurosurgical impairment of the blood–brain barrier. This study is a pilot research of cefoperazone/sulbactam using in neurosurgical individuals, However, it needs to be confirmed by further large-scale studies.

## Background

Among several major Gram-negative bacteria causing all nosocomial infections, the increased incidences of Acinetobacter spp. and Pseudomonas aeruginosa are significant, and the rate of resistance is high.

At the same time, an epidemiological survey on hospital-acquired infections in Beijing Tiantan Hospital, Capital Medical University, also showed that, among patients with intracranial infections after craniotomy, the most common pathogenic bacteria are *Acinetobacter baumannii* and *Pseudomonas aeruginosa*, followed by *Enterobacteriaceae,* such as *Escherichia coli* and *Klebsiella pneumoniae* [1]. Cefoperazone/sulbactam provides good antibacterial activity against *Acinetobacter baumannii* and *Pseudomonas aeruginosa*. Moreover, sulbactam plays an important role in overcoming an emerging worldwide problem as it is effective against *Acinetobacter baumannii* [2-5]. However, the blood–brain barrier limits the efficacy of cefoperazone/sulbactam in intracranial infection. In light of this, the present study was designed to explore whether there is an increased CSF penetration of cefoperazone/sulbactam into the cerebrospinal fluid (CSF) increases after the blood–brain barrier is impaired following craniotomy, and whether extended infusion time affects drug concentrations.

## Methods

The study was approved by the Research Ethics Committee in Tiantan Hospital, Capital Medical University (Beijing, China). Written informed consent was obtained from all patients or their healthcare surrogates prior to enrollment in the study.

### Inclusion criteria

Neurosurgical patients (over 18 years old) with an indwelling ventricular drainage pipe after neurological surgery who were treated with cefoperazone/sulbactam for prevention of intracranial infection were eligible. Patients with histories of heart, pulmonary, liver, and renal dysfunctions were excluded.

### Administration of cefoperazone/sulbactam and specimen collection

Cefoperazone/sulbactam (Sulperazon, Pfizer, New York, USA) in 1.5-g ampoules with cefoperazone 1.0 g/sulbactam 0.5g was given to all included patients. All the patients were given cefoperazone/sulbactam 3.0 g in 50-mL saline by intravenous injection for 3 hours every 6 hours after craniotomy. 1.5mL of venous blood and 1.5 mL of CSF were collected before the start of drug administration and at Hour 1, 2, 3, 4, 6, 12, 15, 16, 18 h after administration. The specimens were centrifuged at a speed of 3500 r/min for 5 min. Then, the supernatant was collected and stored at −70°C for uniform testing.

### Measurement of cefoperazone and sulbactam concentrations

Cefoperazone and sulbactam concentrations were measured with liquid chromatography mass spectrometry (LC-MS/MS).

#### Equipment

The tools in our study included an Agilent 1200 liquid chromatograph (quaternary low pressure gradient pump, automatic on-line degasser, autosampler, column oven, and diode array detector, Agilent Technologies, Inc., Waldbronn, Germany), Chem Station workstations (Agilent Technologies, Inc., Waldbronn, Germany), Thermo LTQ XL mass spectrometer (Thermo Fisher Scientific, Inc., San Jose, CA, USA), OHAUS CP214 electronic balance (Ohaus Corporation. Shanghai, China), Mettler Toledo Seven Easy pH meter(Mettler-Toledo International Inc. Shanghai, China), and KH-500 ultrasonic cleaner (Kunshan Wo-invasive Ultrasound Instruments, Inc. Kunshan, Jiangsu, China) were used.

#### Determination of the standard curve

The cefoperazone sodium reference substance (Pfizer) was dissolved in double-distilled water (1004 mg/L). The sulbactam sodium reference substance was also dissolved in double-distilled water and the concentration was 402 mg/L. The appropriate amounts of stock solution of cefoperazone were taken and diluted with water to 0.1 mg/L, 0.5 mg/L, 1.0 mg/L, 2.5 mg/L, 5.0 mg/L, 10.0 mg/L, and 20.0 mg/L as reference solutions. The appropriate amounts of stock solution of sulbactam were taken and diluted with water to 0.02 mg/L, 0.1 mg/L, 0.2 mg/L, 0.5 mg/L, 1.0 mg/L, 2.0 mg/L, and 4.0 mg/L as reference solutions. 100 μL of the reference solution was drawn into a 1.5-mL centrifuge tube, and dried in a stream of nitrogen. The 200-μL blank plasma was added into the tube and dubbed in standard drug-containing plasma after swirling and mixing. Subsequently, 100 μL of chloramphenicol solution (50 mg/L) of the internal standard (IS) and 50 μl of HCL (1.0 mol/L) were added and mixed, and then, ethyl acetate was used to extract the drug. After centrifugation (16000 g for 5 min), the supernatant was dried and the residue was dissolved in a 200-μL mobile phase and centrifuged at 8000 g for 5 min. The supernatant was filtered with a 0.2-μm membrane and analyzed by LC-MS/MS. The CSF specimen was handled in the same process. The ratio (Y) of the cefoperazone-sulbactam peak area (AS) and to the IS peak area (AI) was calculated, and the cefoperazone-sulbactam plasma concentration (X) was weighted (1/x2) for regression calculation. The correlation coefficient R of the serum cefoperazone concentration and the serum sulbactam was 0.9958 and 0.9913, respectively. The respective correlation coefficient R of CSF cefoperazone concentration and CSF sulbactam concentration was 0.9921 and 0.9532.

#### Sample processing and measurement

The samples were thawed to room temperature and vortex mixed. The supernatant was analyzed by LC-MS/MS, and the peak area was recorded. The peak area of the main component in the sample solution was brought into the working standard curve to calculate cefoperazone/sulbactam content in each sample solution.

### Statistical processing and data analysis

All the data were analyzed by the Microsoft Office Excel 2007. Mean serum and CSF cefoperazone and sulbactam concentration was calculated at each time point and expressed as means±standard deviation (x±SD). The mean value of CSF penetration of cefoperazone and sulbactam was also expressed as means±standard deviation (x±SD). The drug concentration-time curves were drawn; The area under the curves were further calculated. The relationships of cefoperazone/sulbactam concentrations in CSF and serum at each time point with the MIC50 and MIC90 of corresponding bacteria were examined.

## Results

From July 2011 to November 2011, 8 cases (6 males and 2 females) were enrolled. Their mean age was 53±15 years (39 to 77 years), and their average weight was 72.5±9.4 kg (60 to 84 kg). Of the 8 included patients, 3 cases underwent meningioma resection, 3 cases of glioma resection, 1 case of hematoma clearance of hypertensive intracerebral hemorrhage, and shunt surgery after carotid artery aneurysm intervention, respectively.

Table 1 shows the baseline data. The average serum and CSF concentrations of cefoperazone and sulbactam at all time point was given in Table 2.

**Table 1:** **Characteristics of enrolled patients**

Case	Diagnosis	Drainage tube position	Creatinine (μmol/L)	Administration time	Antibiotics before administration
1	right frontal meningioma	operative field into intraventricular	57	within 3 h after surgery	
2	left basal ganglia hemorrhage dissection	ventricles	57	10 d after surgery	piperacillin/sulbactam (4:1)
3	right frontotemporal glioma	operative field into intraventricular	46	2 d after surgery	piperacillin/sulbactam (4:1)
4	right frontal glioma	operative field into intraventricular	49	within 3 h after surgery	
5	right triangle intraventricular meningioma	operative field into intraventricular	94	within 3 h after surgery	piperacillin/sulbactam (4:1)
6	Internal carotid artery aneurysm after interventional treatment, intraventricular hemorrhage	ventricles	26	within 3 h after surgery	
7	left frontal glioma	operative field into intraventricular	57	within 3 h after surgery	
8	Foramen magnum meningioma	ventricles	37	within 7 d after surgery

**Table 2:** **Average serum and CSF concentrations of cefoperazone and sulbactam at every time point (mg/L)**

Time	Cefoperazone(x±sd)(n)(range)		Sulbactam(x±sd)(n)(range)	
	Serum	Cerebrospinal fluid	Serum	Cerebrospinal fluid
Prior to administration	1.17±0.92(3) (0.51-2.22)	0.30±0.13(2) (0.2,0.39)	3.22±4.23(4) (0.2-9.4)	4.00±4.27(4) (0.38-10.07)
1	191.70±108.76(4) (33.45-281.39)	9.64±15.90(3) (0.44-28)	54.21±59.62(4) (15.29-143.09)	3.92±8.14(5) (0.07-18.48)
2	237.54±336.72(6) (14.84-891.77)	7.13±14.95(5) (0.28-33.87)	66.52±80.38(6) (3.7-223.15)	3.64±5.24(6) (0.18-14.07)
3	143.88±133.86(6) (30.21-343.82)	13.75±31.59(6) (0.05-78.21)	25.31±7.51(5) (13.19-33.27)	3.91±4.90(6) (0.18-13.33)
4	109.01±158.83(4) (21.23-346.98)	39.22±75.55(4) (1.05-152.55)	10.24±10.18(4) (2.23-25.05)	6.24±8.35(4) (0.55-18.59)
6	70.22±93.83(8) (3.42-282.65)	14.37±25.33(8) (0.38-75.76)	8.13±11.75(8) (0.1-30.27)	3.82±2.92(8) (0.42-10.01)
12	69.23±81.83(8) (3.62-192.77)	10.19±12.84(8) (0.33-36.38)	6.49±12.12(8) (0.32-36.29)	3.28±3.02(8) (0.33-9.56)
15	154.62±203.35(4) (35.58-457.64)	23.93±43.77(4) (0.32-89.55)	30.57±20.50(4) (13.61-56.46)	3.25±2.10(4) (0.27-5)
16	124.94±153.58(5) (22.17-386.35)	20.43±29.08(5) (0.62-68.35)	11.51±9.40(5) (4.69-27.66)	5.66±5.32(5) (0.41-13.65)
18	72.49±90.14(3) (3.28-174.42)	3.42±2.95(4) (0.28-7.21)	3.55±2.63(3) (0.61-5.67)	1.32±1.55(5) (0.16-3.86)

CSF penetration measured by the ratio of peak concentrations (CSF/serum) was 8.6%±7.2% for cefoperazone and 13.5%±11.9% for sulbactam, CSF penetration measured by the ratio of trough concentrations (CSF/serum) was 13.4%±5.3% for cefoperazone and 106.5%±87.5% for sulbactam. In addition, the CSF penetration can be evaluated by the ratio of the area under the curve (AUC) of CSF and serum, and the ratio was 14.5% for cefoperazone and 22.6% for sulbactam in the group. If the CSF penetration was evaluated by the ratio of average peak concentration (cerebrospinal fluid/serum), the penetration of cefoperazone and sulbactam was 16.0% and 13.9%, respectively. Table 3 shows the CSF penetration of each individual.

**Table 3:** **CSF penetration of individual patient**

Penetration	Peak cencentration (cefoperazone)	Trough concentration (cefoperazone)	Peak concentration (sulbactam)	Trough concentration (sulbactam)
1	13.0%	3.8%	9.4%	103.0%
2	6.5%	14.9%	22.7%	149.0%
3	3.7%	18.1%	6.9%	140.4%
4	1.3%	17.5%	1.6%	68.8%
5	17.1%	18.9%	8.3%	54.2%
6	5.9%	10.0%	16.9%	288.0%
7	20.0%	14.4%	37.6%	26.3%
8	1.4%	9.2%	4.5%	22.5%
X±SD	8.6%±7.2%	13.4%±5.3%	13.5%±11.9%	106.5%±87.5%

**Figure 1:**
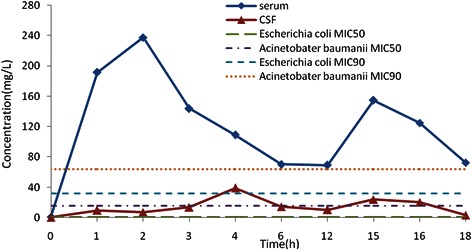
**Cefoperazone serum and CSF concentration-time curve in the group.** Square dotted lines represent cefoperazone against Acinetobacter baumannii and Pseudomonas aeruginosa MIC90, T>MIC% was greater than 50%. Triangular dotted lines represent cefoperazone against Acinetobacter baumannii and Escherichia coli MIC50. T>MIC% was almost 100%(Escherichia coli MIC50) and T>MIC% was more than 50%(Acinetobacter baumanniiMIC50).

**Figure 2:**
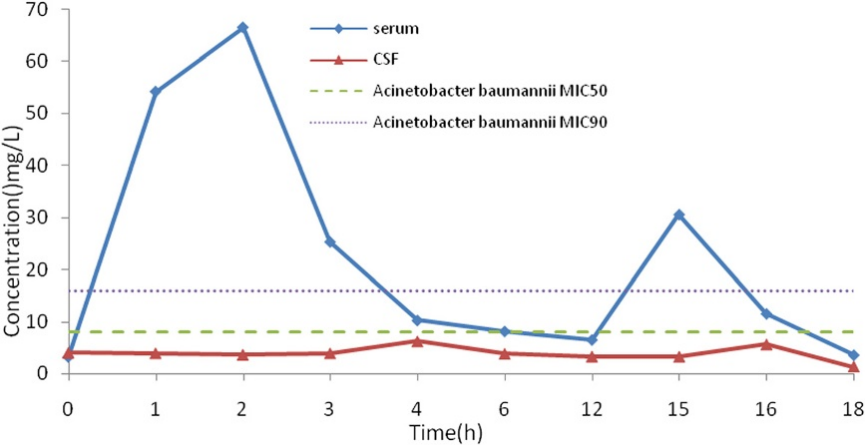
**Sulbactam serum concentration-time curve in the group.** Square dotted lines represent sulbactam against Acinetobacter baumanniiMIC50 and MIC90, most areas under the curve were beyond the MIC90, T>MIC% was greater than 50%. Triangular dotted lines represent sulbactam against Acinetobacter baumanniiMIC50, all the area under the curve were blow the MIC50.

**Figure 3:**
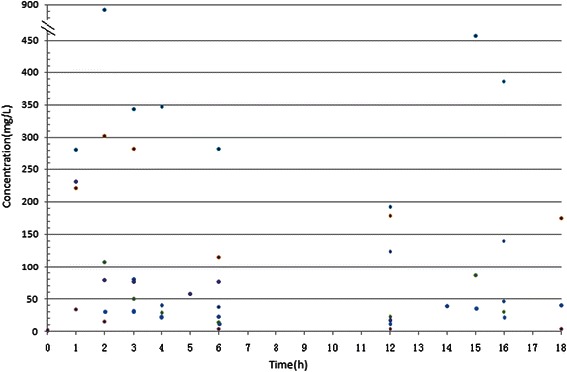
**A scatter plot of serum concentration-time for Cefoperazone.** The straight line represents the cefoperazone against Acinetobacter baumannii MIC50, we can see the most scatter plots are beyond the line (32/62).

**Figure 4:**
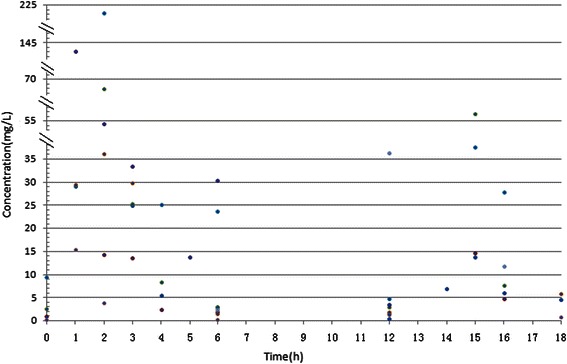
**A scatter plot of serum concentration-time for Sulbactam.** The straight line represents the sulbactam against Acinetobacter baumannii MIC50, we can see the most scatter plots are beyond the line (27/53).

**Figure 5:**
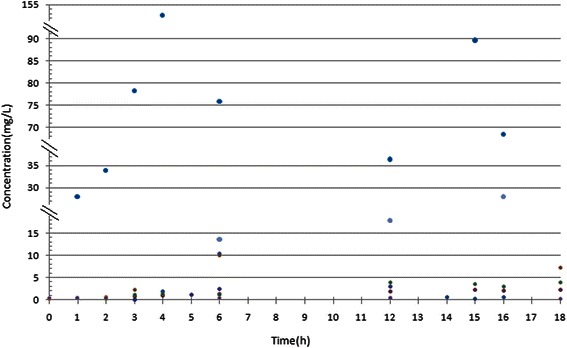
**A scatter plot of CSF concentration-time for Cefoperazone.**

**Figure 6:**
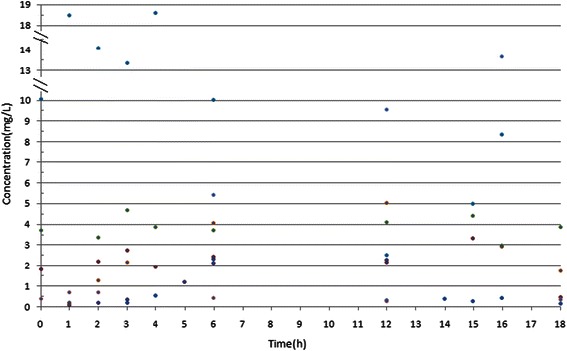
**A scatter plot of CSF concentration-time for Sulbactam.**

Figure 1 is the cefoperazone serum and CSF concentration-time curve in the group. With MIC_90_ for cefoperazone/sulbactam against *Acinetobacter baumannii* and *Pseudomonas aeruginosa* at 64 mg/L as an example (cefoperazone concentration marked), T>MIC% was greater than 50% [6]. With MIC_50_ of 16 mg/L for cefoperazone/sulbactam against *Acinetobacter baumannii* and *Pseudomonas aeruginosa* as an example, T>MIC% was about 50% [6]. With MIC_50_ of cefoperazone against *Enterobactercloacae* at 2 mg/L, *Escherichia coli* at 1 mg/L, *Klebsiella pneumoniae*at 0.5 mg/L as examples, T>MIC% was close to 100% [7]. With MIC_90_ of 16 mg/L for sulbactam against *Acinetobacter baumannii* as an example, T>MIC% was greater than 50% (Figure 2). Figure 2 shows the sulbactam CSF concentration-time curve in the group. The sulbactam concentration did not reach the level of MIC_50_of 8 mg/L for *Acinetobacter baumannii* [8,9].The relationship between cefoperazone concentrations at different time points and MIC_50_ was presented in Figure 3. There are 51.6% (32/62) of the cefoperazone concentrations at different time points over MIC_50_. Figure 4 described the relationship of sulbactam concentrations at different time points with MIC_50,_There are 50.9% (27/53) of the sulbactam concentrations at different time points over MIC_50_. A scatter plot of CSF concentration-time for Cefoperazone and Sulbactam were described in Figure 5 and Figure 6, respectively.

## Discussion

PK/PD studies in recent years have shown that extension of the single-dose infusion time can improve the efficacy of time-dependent antibiotics. The results of the present research consistently suggested that, if cefoperazone/sulbactam single infusion time was extended to 3 hours, the serum drug concentration achieved the PK/PD standard of T>MIC% greater than 50% (MIC_90_64 mg/L). However, due to the development of drug resistance, the bacteria’s MIC is generally high. Consequently, it is very difficult to achieve this PK/PD standard in the CSF, and a higher dose might be needed to treat intracranial infections. Taking into account the safety of the drug, a daily dose of 4 g daily dose of sulbactam was administered in the study. However, some papers recommended a daily sulbactam dose 6 g for the treatment of hospital-acquired Gram-negative bacterial infections, especially carbapenem-resistant Acinetobacter baumannii [10]. On the other hand, despite the presence of the blood–brain barrier, drug doses cannot be increased without limitations. For intracranial infections caused by highly resistant bacteria, such as Acinetobacter baumannii, a special methods of drug administration, including extending the single infusion time while giving larger doses, local administration within the ventricles or subarachnoid space may need to be considered on the basis of strengthening comprehensive supportive care. Still, more studies are required to answer the question whether the specific method and dose administered topically can improve the therapeutic effect and how to avoid drug adverse effects.

It has been previously found that there was an increase CSF penetration of vancomycin and cefepime after the destruction of the blood–brain barrier following neurosurgical procedures [11-14]. CSF penetration of cefoperazone in the treatment of adult meningitis has been reported to be 6.4% (concentration ratio) [15]. It is related to the severity of destruction in the blood–brain barrier by inflammation, and it was lower than the result found in the present study (14.5%). For infant meningitis [16], CSF concentration of sulbactam is up to 5.5±8.7 mg/L when using ampicillin/sulbactam (400 mg/kg per day for ampicillin, 50 mg/kg per day for sulbactam), which is lower than the present result (6.24±8.35 mg/L). For patients with negative CSF cultures, CSF penetration is less than 17% (concentration ratio), which is similar to the 13.9% in the present study. Compared with other reports [16,17], the CSF drug concentration was higher in the extending group. Although the study is a pilot research, the trend illustrate the higher concentration might be got with extending administration, so when it is not effective of cefoperazone/sulbactam in bacterial meningitis, the administration method might be another choice.

Limitations of this experiment included that it was very difficult to get clinical CSF specimens, as specimens could not be stored for a long period, and the number of enrolled patients was low. Thus, there were no clear relationships between drug concentration fluctuations and patients’ age, weight, lesions, surgical characteristics, CSF drainage, and administration time. These factors, coupled with time error of specimen collection, and different specimen storage time, resulted in the large standard deviation of the research data being too large. However, in previous relevant literature, large standard deviations were also observed. In my opinion, such common great variation might be caused by many factors, particularly different CSF concentration after the various damage of blood–brain barrier [16,18,19]. In addition, all the individuals were given the same doses of cefoperazone/sulbactam in the study, not according to the weight of each patient, and the different ventriclular or operative field into intraventricular drainages, the factors all may effected the standard deviations were large.

The results of this study suggest that if the single intravenous administration time of cefoperazone/sulbactam is 3 hours, the serum concentrations can meet the PK/PD standard (T>MIC% greater than 50%) for common, highly resistant bacteria such as *Acinetobacter baumannii* and *Pseudomonas aeruginosa* (MIC_90_ reached 64 mg/L). Furthermore, extending administration time can improve the PK/PD indices of the two drugs, and further improve clinical efficacy. In addition, destruction of the blood–brain barrier after craniotomy can increase the CSF concentration to a certain extent. However, for intracranial infections caused by highly resistant bacteria, comprehensive treatment may be needed with a higher dosage of antibiotics, otherwise the antibiotics may need to be given through the ventricle and subarachnoid topically.

## Conclusions

The CSF penetration of cefoperazone/sulbactam may be enhanced after neurosurgical impairment of the blood–brain barrier. It may be needed enhance doses of sulbactam for reaching the effective concentrations in CSF (e.g. MIC50/MIC90 of Acinetobacter baumannii).This study is a pilot research of cefoperazone/sulbactam using in neurosurgical individuals, However, it needs to be confirmed by further large-scale studies.

## Abbreviations

CSF: Cerebrospinal fluid
AUC: Area under the curve
MIC: Minimum inhibitory concentration
PK/PD: Pharmacokinetic/Pharmacodynamic
SD: Standard deviation
